# CD206^+^/MHCII^−^ macrophage accumulation at nerve injury site correlates with attenuation of allodynia in TASTPM mouse model of Alzheimer's disease

**DOI:** 10.1016/j.bbih.2022.100548

**Published:** 2022-11-01

**Authors:** Rita Silva, George Sideris-Lampretsas, Sarah Fox, Lynda Zeboudj, Marzia Malcangio

**Affiliations:** Wolfson Centre for Age Related Diseases, King's College London, London, SE1 1UL, UK

**Keywords:** Neuropathic pain, Alzheimer's disease, Opioids, Sciatic nerve, Macrophage, Monocyte

## Abstract

Chronic pain is undertreated in people with Alzheimer's disease (AD) and better understanding of the underlying mechanisms of chronic pain in this neurodegenerative disease is essential. Neuropathic pain and AD share a significant involvement of the peripheral immune system. Therefore, we examined the development of nerve injury-induced allodynia in TASTPM (APPsweXPS1.M146V) mice and assessed monocytes/macrophages at injury site. TASTPM developed partial allodynia compared to WT at days 7, 14 and 21 days after injury, and showed complete allodynia only after treatment with naloxone methiodide, a peripheralized opioid receptor antagonist. Since macrophages are one of the sources of endogenous opioids in the periphery, we examined macrophage infiltration at injury site and observed that CD206^+^/MHCII^−^ cells were more numerous in TASTPM than WT. Accordingly, circulating TASTPM Ly6C^high^ (classical) monocytes, which are pro-inflammatory and infiltrate at the site of injury, were less abundant than in WT. In *in vitro* experiments, TASTPM bone marrow-derived macrophages showed efficient phagocytosis of myelin extracts containing amyloid precursor protein, acquired CD206^+^/MHCII^−^ phenotype, upregulated mRNA expression of proenkephalin (*PENK*) and accumulated enkephalins in culture media. These data suggest that in TASTPM nerve-injured mice, infiltrating macrophages which derive from circulating monocytes and may contain amyloid fragments, acquire M2-like phenotype after myelin engulfment, and release enkephalins which are likely to inhibit nociceptive neuron activity via activation of opioid receptors.

## Highlights

• Mechanical allodynia is attenuated and linked with higher peripheral opioidergic tone in TASTPM model of Alzheimer's Disease.
• Higher number of macrophages and CD206^+^/MHCII^−^ macrophage population accumulate at nerve injury site in TASTPM mice.
• Fewer Ly6Chigh monocytes circulate in TASTPM bloodstream.
• TASTPM macrophage exposure to myelin and amyloid-beta induces CD206^+^/MHCII^-^ phenotype and enkephalin release.

## Abbreviations

**AD –**: Alzheimer's Disease
**APP/Abeta**–: Amyloid Precursor Protein/Amyloid beta
**BMDMs –**: Bone Marrow Derived Macrophages
**CCR2**–: C–C Chemokine receptor type 2
**CD11b**–: Cluster of Differentiation 11B, also encoded by the *Itgam* gene
**CD206**–: Cluster of Differentiation 206, Mannose Receptor C type 1
**FACS**–: Fluorescence Activated Cell Sorting
**FMO**–: Fluorescence Minus One
**IL1β**: Interleukin 1-beta
**IL6**–: Interleukin 6
**MFI**–: Mean Fluorescence Intensity
**MHC II**–: Major Histocompatibility Complex Class II receptor
**Ly6C**–: Lymphocyte antigen 6 complex
**pPENK**–: Pre-proenkephalin
**PENK –**: Proenkephalin
**PWT**–: Paw Withdrawal Threshold
**SNI**–: Spared Nerve Injury
**TNFα**–: Tumour necrosis factor alpha
**TGFb**–: Transforming Growth Factor Beta

## 1 Introduction

The co-morbidity of Alzheimer's disease (AD) and chronic pain in the growing ageing population needs to be addressed if proper pain control is to be achieved in people with AD (Corbett et al., 2012; Cao et al., 2019). This is a challenging proposal due to limited capacity of self-report by these individuals and lack of valid and reliable assessment tools. As a result, pain in AD patients remains undertreated and misdiagnosed highlighting an urgent need to identify mechanisms of chronic pain in AD in preclinical settings and provide tailored strategies for treatment of pain in AD patients (Lawn et al., 2021).

Pain processing is altered in people with AD compared with healthy controls and imaging studies indicate greater emotional activity, diminished placebo response, and increased susceptibility to opioid-related harm (Lawn et al., 2021). Besides neuronal pathology, a prominent feature of the AD brain is significant activation of immunocompetent cells. Indeed, brain microglia have recently received intense attention as they express genes associated with sporadic AD (Heneka et al., 2015). Microglial activity is driven by the close association with amyloid plaques and it is also influenced by alteration of the peripheral immune system with implications for cognitive function and clinical stage of AD (Heneka et al., 2015; Bettcher et al., 2021). Indeed, monocytes/macrophages of AD patients display functional changes such as poor differentiation *in vitro*, weak phagocytic properties and undergo apoptosis after exposure to Aβ (Fiala et al., 2005). More specifically, recent studies have showed that monocytes/macrophages differentiation and activation appear to depend on the various stages of AD. In fact, monocytes from mild cognitive impairment (MCI) patients show higher phagocytic activity compared to other stages of the pathology (Munawara et al., 2021).

Chronic pain and AD share a significant involvement of the immune system, as both microglia in the CNS and monocytes/macrophages in the periphery play significant mechanistic roles in chronic pain. Specifically in neuropathic pain following peripheral nerve injury, spinal cord microglia respond to increased neuronal activity at the first pain synapse by changing morphology and releasing mediators that sensitise neurons (Clark et al., 2007; Tsuda et al., 2003; Guan et al., 2016; Gu et al., 2016). At the site of nerve injury and in dorsal root ganglia, classical monocytes infiltrate nervous tissues and macrophages engraft in the pool of resident macrophages (Ydens et al., 2020). Within the first few days after injury pro-inflammatory macrophages (M1-like phenotype) facilitate nociceptive transmission (Silva et al., 2021; Barclay et al., 2007) and for instance, angiotensin-receptor antagonists exert anti-allodynic effects by blocking angiotensin-2 receptors in macrophages 8 days after injury (Shepherd et al., 2018). However, macrophages at the site of injury also play a critical role in nerve regeneration mechanisms and display M2-like phenotype, especially at distal site from the injury where they can be found weeks after injury (Kalinski et al., 2020; Chen et al., 2015). Relevantly, M2-like macrophages can exert anti-allodynic effects when injected locally at the site of nerve injury since they release opioids peptides (Celik et al., 2016; Pannell et al., 2016).

Current evidence in AD transgenic mice resembles some aspects of the clinical settings including amyloid deposits and neuroinflammation in the brain, amyloid fragments in blood monocytes and monocytopenia, and cognitive and sensory deficits (Aman et al., 2016; Naert and Rivest, 2012). For instance, 6 month-old TASTPM mice (APPsweXPS1.M146V) display i) age-dependent increase of thermal thresholds in association with amyloid beta plaques, ii) microgliosis and astrocytosis in pain related areas of the brain and spinal cord iii) age-dependent decrease of amyloid peptides in plasma, iv) increase of amyloid peptides in monocytes and v) cognitive impairment associated with increased presence of amyloid plaques (Aman et al., 2016; Halle et al., 2015).

Therefore, we used TASTPM mice to evaluate the development of neuropathic pain and test the hypothesis that immune-mediated mechanisms are at play in this model of AD.

## 2 Material and methods

### 2.1 Animals

Experiments were performed on in 6–7 months old adult male and female (1:1) heterozygous double-mutant TASTPM transgenic mice. TASTPM mice (GlaxoSmithKline) express human mutant amyloid precursor protein (hAPP695swe) and presenilin-1 (M146V) under the control of the neuron specific Thy-1 promoter on a C57BL/6 background. Age and sex-matched C57BL/6 obtained from Charles River Laboratories were used as controls (WT). All animals were housed in the Biological Services Unit, King's College London and maintained in 12 h day/night cycle with access to food and water *ad libitum*. All experiments were conducted according to the United Kingdom Animals (Scientific Procedures) Act 1986 and following the guidelines of the Committee for Research and Ethical Issues of the International Association for the Study of Pain (IASP).

### 2.2 Behavioural testing

Static mechanical withdrawal thresholds were assessed by application of calibrated von Frey monofilaments (0.008–1.0 g) to the hind paw plantar surface. Paw withdrawal threshold (PWT) (50%) was determined by increasing or decreasing stimulus and evaluated using the Dixon “up-down” method (Dixon, 1980). Mice were placed individually and acclimatized up to 30 min, prior testing, in acrylic cubicles on a wire mesh grid. Testing started with the application of a 0.07 g filament and each paw was assessed alternately between application of increasing stimulus intensity until a withdrawal response was achieved or application of 1.0 g filament failed to induce a response, in order to avoid tissue damage. All experiments were performed blind.

### 2.3 Induction of neuropathy

Mice underwent a SNI surgery under isoflurane anaesthesia (Decosterd and Woolf, 2000). Briefly, the skin and muscle of the left thigh were incised to expose the sciatic nerve and its three terminal branches. The common peroneal and tibial nerves were identified and the distal nerve stump was removed, while the sural nerve was left intact (Decosterd and Woolf, 2000). In sham-injured mice, sciatic nerve was exposed but not excised. Mechanical thresholds (PWTs) were examined 3 consecutives days prior to and on days 7, 14 and 21 after surgery.

### 2.4 Naloxone administration

Naloxone hydrochloride (1 mg/kg; Sigma-Aldrich) and Naloxone methiodide (1 mg/kg; Sigma-Aldrich) were dissolved in sterile saline. Either naloxone hydrochloride, naloxone methiodide and saline were administered intraperitoneally in injured TASTPM and WT animals. Baseline paw withdrawal thresholds were recorded before naloxone administration and assessed 30 min after drug administration.

### 2.5 Processing and flow cytometry of sciatic nerve

Mice were administered an intraperitoneal overdose of pentobarbital (Euthatal; Merial, Duluth, GA) and perfused with phosphate-buffer saline (PBS) to prevent from peripheral blood contamination. The sciatic nerve was exposed and injury site was identified. One centimetre of the injury site (containing the injury site and proximal segment close to injury site) was cut and placed into a Petri dish containing F12 medium (Gibco, New York, NY). Samples were transferred into 1.5 mL Eppendorf tube and processed using 50 μl of digestion mix: F12 with 0.125% collagenase type IV (Sigma-Aldrich); 3 mg/mL dispase II (Roche) and 200 U/mL DNAse I (Roche). Tissue suspension was placed at 37 °C for 45 min with gentle agitation (≤300 RPM). Cell suspension underwent mechanical dissociation and later incubated for an additional period of 15 min at 37 °C with gentle agitation (≤300 RPM). Samples were centrifuged twice at 300×*g* for 5 min at 4 °C and pellet resuspended in 500 μl PBS after first centrifugation and in 100 μl PBS in the last centrifugation. Cell suspensions were incubated with Zombie NIR™ Fixable Viability kit (BioLegend) for 15 min and cell suspensions were centrifuged for 5 min at 300×*g* at 4 °C. Suspensions were further incubated on ice for 15 min with anti-mouse CD16/CD32 (Clone 93; BioLegend, RRID:AB_312800) to block Fc receptors in 100 μl FACS buffer (filter-sterilized PBS with 0.5% BSA (Sigma Aldrich) and 2 mM EDTA (ThermoFisher)) and followed by incubation for 30 min, on ice, with a mix of fluorochrome-conjugated anti-mouse antibodies protected from light: CD11b-BV421 (Clone M1/70, BioLegend, RRID:AB_10897942), F4/80-APC (Clone BM8, BioLegend, RRID: AB_893481), CD206-PE (Clone C068C2, BioLegend, RRID: AB_10896421) and MHCII-PerCPCy5.5 (Clone AF6-120.1, BioLegend, RRID: AB_1953398). Following centrifugation, the staining was washed and cells were diluted in 200 μl CellFIX™ tissue processing reagent (BD Biosciences) containing 20 μl of Precision Count Beads™ (BioLegend) before being analysed through a LSRFortessa™ cell Analyzer (BD Bioscience). Unstained cells and single staining control beads (UltraComp eBeads™ Compensation Beads, ThermoFisher) were used for compensation. Fluorescence-minus one (FMO) controls were recorded and used for gating. Raw data was analysed with FlowJo software (v10.7.1, BD Biosciences).

### 2.6 Processing and flow cytometry of peripheral blood

Mice were administered an intraperitoneal overdose of pentobarbital (Euthatal; Merial, Duluth, GA) and blood was removed using a 23G needle attached to a 1 mL syringe. A cardiac puncture was performed and approximately 0.5 mL of blood was drawn from each animal. The blood sample was transferred into EDTA tubes (ThermoFisher). Blood samples (50 μl) were transferred onto a 96-well v-bottom plate and immediately incubated with Zombie NIR™ Fixable Viability kit (BioLegend) for 15 min and cell suspensions were centrifuged for 5 min at 300×*g* at 4 °C. The pellet was re-suspended and further incubated on ice for 15 min with anti-mouse CD16/CD32 (Clone 93; BioLegend, RRID:AB_312800) to block Fc receptors in FACS and followed by incubation for 30 min, on ice, with a mix of fluorochrome-conjugated anti-mouse antibodies protected from light: CD11b-APC (Clone M1/70, eBioscience, RRID: AB_469343), Ly6C-FITC (Clone HK1.4, BioLegend, RRID: AB_1186134) and Ly6G-PE (Clone IA8-Ly6G, eBioscience, RRID: AB_2572720). After centrifugation for 5 min at 300×*g* at 4 °C, the pellet was resuspended in red blood lysing buffer (1:10) (BD™ FACS Lysing Solution, BD Biosciences) in the dark at room temperature for 15 min and followed by centrifugation for 5 min at 300×*g* at 4 °C. The previous red blood lysing step was repeated one more time for 10 min (incubation period) to achieve successful red blood cells lysis. Following centrifugations, pellet was resuspended in in 200 μl CellFIX™ tissue processing reagent Buffer (BD Biosciences) containing 20 μl of Precision Count Beads™ (BioLegend) before being analysed through a LSRFortessa™ cell Analyzer (BD Bioscience). Unstained cells and single staining control beads (UltraComp eBeads™ Compensation Beads, ThermoFisher) were used for compensation. Fluorescence-minus one (FMO) controls were recorded and used for gating. Raw data was analysed with FlowJo software (v10.7.1, BD Biosciences).

### 2.7 Myelin extract purification

Myelin extracts were obtained according to Erwig et al. (2019). Briefly, freshly dissected brains from WT and TASTPM mice were homogenized separately with 5 mL 0.32 M sucrose (Sigma Aldrich) in precooled Beckman centrifuge tubes using a Dounce homogenizer. To an additional Beckman centrifuge tube, 6 mL 0.85 M sucrose solution was added and the homogenate was layered carefully on top. Tube was centrifuged 75,000×*g* for 30 min at 4 °C. The interface obtained was collected and washed twice with ddH_2_O. The process is repeated twice and after several ultracentrifugations, the pellet was re-suspended in PBS, transferred into a sterile tube and stored at −80 °C.

### 2.8 Human APP/Abeta detection in myelin extracts by Western blot

WT and TASTPM myelin extracts were homogenized in RIPA buffer (Sigma Aldrich) containing protease inhibitor cocktail tablets (Roche) and maintained on ice for 15 min. Following incubation, lysates were centrifuged at 13000 RPM for 20 min at 4 °C. Supernatant was removed and protein concentration was determined using the Pierce™ BCA Protein Assay kit (Invitrogen), according to manufacturers’ instructions.

Equal protein concentrations (20 μg) were added to 4x Laemmli sample buffer, boiled for 5 min, and subjected to a 10% SDS-PAGE gel. Wet transfer was performed into PVDF membranes using the Trans-Blot Turbo Transfer System (BioRad). The PVDF membrane was immersed in warmed PBS for 5 min, according to antibody manufacturer's instructions. The membrane was incubated with 5% non-fat-dried milk in TBS-T (50 mM Tris-HCl, pH 7.6, 150 mM NaCl, 0.1% Tween 20) for 1 h at room temperature and followed by incubation overnight with primary antibodies: anti-mouse β-amyloid (6E10, 1:1000, BioLegend, RRID: AB_2728527), anti-mouse NeuN (Cell Signaling, 1:1000, RRID AB_2630395) and anti-mouse GAPDH (6C5, Abcam, 1:1000, RRID: AB_2107448) at 4 °C under constant gentle shaking. Results were visualized with horseradish peroxidase-coupled anti-mouse or anti-rabbit immunoglobulin (1:1000, Dako) using enhanced chemiluminescence detection reagents (ECL, EMD Millipore). Protein band densities were visualized by ImageLab™ (BioRad).

### 2.9 Myelin extracts labelling

WT and TASTPM myelin extracts (20 μg) were labelled with CellTrace™ Far Red Cell Proliferation Kit (Invitrogen) according to manufacturer's instructions. Briefly, myelin extracts were incubated with 1 μl Cell Trace for 20 min at 37 °C protected from light. To remove any free dye remaining from the solution, five times more the original volume was added to the samples and incubated for 5 min. Suspensions were centrifuged for 5 min at 300×*g* and re-suspended in PBS. Following re-suspending, samples were incubated for 10 min at 37 °C before use.

### 2.10 Bone marrow derived macrophages (BMDMs) culture and myelin extract stimulation

Hematopoietic stem cells were isolated from the femur and tibia bones from WT and TASTPM mice and allowed to differentiate into macrophages for 7 days at 37 °C 95%O_2_/5% CO_2_ conditions in high glucose Dulbecco's modified Eagle Media (DMEM) supplemented with 10% heat inactivated fetal bovine serum (FBS, Sigma Aldrich), 1% penicillin/streptomycin (P/S, ThermoFisher) and 10% supernatant derived from L929 fibroblast culture as a source of macrophage colony-stimulating factor (Englen et al., 1995) in 100 mm Petri dishes (Thermo Fisher). On day 5, additional 5 mL of fresh medium was added. On day 7, cells were gently dislodged using a cell scrapper and centrifuged for collection (5 min at 300×*g* at 4 °C). Cells were plated at 1 × 10^6^/well density in a 12 well-plate and left to set overnight in DMEM supplemented with 1% FBS (Sigma Aldrich) and 1% P/S (ThermoFisher). The next day cells were incubated with 20 μg APC-labelled myelin extracts from WT and TASTPM mice and incubated for 2 h at 37 °C under 95%O_2_/5% CO_2_ conditions. This experiment was performed 3 independent times, with each replicate of each genotype containing at least 4 animals, unless stated otherwise.

### 2.11 BMDMs flow cytometry

After 2 h, supernatants were collected and remaining adherent cells were washed with PBS. Cells were carefully scrapped using 10 mL of PBS on ice and later centrifuged for 5 min at 300*g* at 4 °C. Cells were incubated with Zombie NIR™ Fixable Viability kit (BioLegend) for 15 min and cell suspensions were centrifuged for 5 min at 300×*g* at 4 °C. The pellet was re-suspended and further incubated on ice for 15 min with anti-mouse CD16/CD32 (Clone 93; BioLegend, RRID:AB_312800) to block Fc receptors in FACS buffer and followed by incubation for 30 min, on ice, with a mix of fluorochrome-conjugated anti-mouse antibodies always protected from light: CD11b-BV421 (Clone M1/70, BioLegend, RRID:AB_10897942), F4/80-PE (Clone BM8, BioLegend, RRID:AB_893498), CD206-PECy7 (Clone C068C2, BioLegend, RRID:AB_2562247) and MHCII-PerCPCy5.5 (Clone AF6-120.1, BioLegend, RRID: AB_1953398). After washes and centrifugations, cells were re-suspended in CellFIX™ tissue processing reagent (BD Biosciences)containing 20 μl of Precision Count Beads™ (Biolegend) before being analysed through a LSRFortessa™ cell Analyzer (BD Bioscience). Unstained cells and single staining control beads (UltraComp eBeads™ Compensation Beads, ThermoFisher) were used for compensation. Fluorescence-minus one (FMO) controls were recorded and used for gating. Raw data was analysed with FlowJo software (v10.7.1, BD Biosciences).

### 2.12 Real time qPCR

Following myelin stimulation, cells were lysed with TRIzol™ Reagent (ThermoFisher) and transferred into a 1.5 mL RNA/DNA-free Eppendorf tube. To the homogenized sample, chloroform (VWR Chemicals) was added to the cell suspension. Tubes were shaken vigorously for 15 s and centrifuged for 15 min at 4 °C at 12000×*g*. Following centrifugation, the upper aqueous phase was collected into a new Eppendorf tube. Isopropanol (ThermoFisher) was added to the tube and the aqueous phase was precipitated by pipetting up and down gently. Samples underwent another centrifugation for 10 min at 4 °C at 12000×*g*. The supernatant was discarded and the RNA was washed through an ethanol gradient (70% and 100% ethanol) followed by centrifugations of 10 min at 4 °C at 12000×*g.* Upon removal of supernatants, RNA pellets were allowed to air dry for 20 min. The RNA pellet was re-suspended in 20 μl of nuclease-free water and RNA quantity/purity was measured using the NanoDrop ND-100 Spectrophotometer (ThermoFischer). For RNA detection, from 400 to 1000 ng of RNA were converted into cDNA using the QuantiTect Reverse Transcription Kit (Qiagen), according to manufacturers’ instructions. qPCR for *PENK* (NM_001002927, Qiagen), *il6 (*NM_031168, Qiagen*), Tnfa* (NM_001159392, Qiagen), *Il1b (*NM_008361, Qiagen), *Ccr2* (NM_009915) and *Tgfb* (NM_011577,Qiagen) was performed using a LightCycler 480 SYBR Green I Master kit (Roche) in a LightCycler 480 (Roche). Duplicate CTs were averaged and were analysed following the 2^−^^ΔΔCT^ method using *18S (*NR_003278, Qiagen) or *Actb (*NM_007393*,* Qiagen*)* as a housekeeper gene.

### 2.13 Immunohistochemistry

For immunohistochemistry, cells (both non- and myelin stimulated BMDMs) were plated at 1 × 10^6^/well in glass coverslips and fixed after myelin stimulation with 4% paraformaldehyde in PBS (ThermoScientific) for 10 min. After fixation, cells were washed three times with PBS containing 0.1% Triton X (Sigma Aldrich). Cells were blocked with PBS containing 2% BSA (Sigma Aldrich) and 0.1 Triton X (Sigma Aldrich) for 2 h at room temperature. Following blocking, cells were incubated with the primary antibody goat anti-rabbit Iba1 (1:1000, Wako, RRID: AB_839504) in PBS containing 0.1% Triton X overnight at 4 °C. Cells were washed several times before incubating with the secondary antibody goat anti-rabbit Alexa Fluor 488 (1:1000, ThermoFisher, RRID: AB_2630356) and Hoechst (1:1000, Invitrogen) for 1 h at room temperature. Coverslips were mounted in SuperFrost Plus microscope slides (VWR) and visualized using a Zeiss LSM710 confocal microscope and images were acquired using the LSM software (Zeiss, UK).

Four coverslips for group were analysed using ImageJ version 1.51 (NIH) to calculate the proportion of Iba1^+^ cells containing myelin to the total number of cells.

### 2.14 ELISA

Enkephalins concentration was determined, through competitive ELISA, using supernatants from non- and myelin stimulated BMDMs from WT and TASTPM mice and was performed according to ELISA manufacturer's instructions (Abbexa, abx156933).

### 2.15 Statistics

Statistical analysis was performed with Graph-Pad Prism (v.9.0.1, Graph-Pad Software). All data are presented as means ± S.E.M. and were analysed using Student's *t*-test (two groups), one-way ANOVA followed by Tukey's multiple comparison test (more than two groups) or two-way ANOVA followed by Tukey test for behavioural data. Differences between means were considered statistically significant when P < 0.05.

## 3 Results

### 3.1 TASTPM develop less severe allodynia than WT (Wild type) mice after peripheral nerve injury

**Fig. 1:**
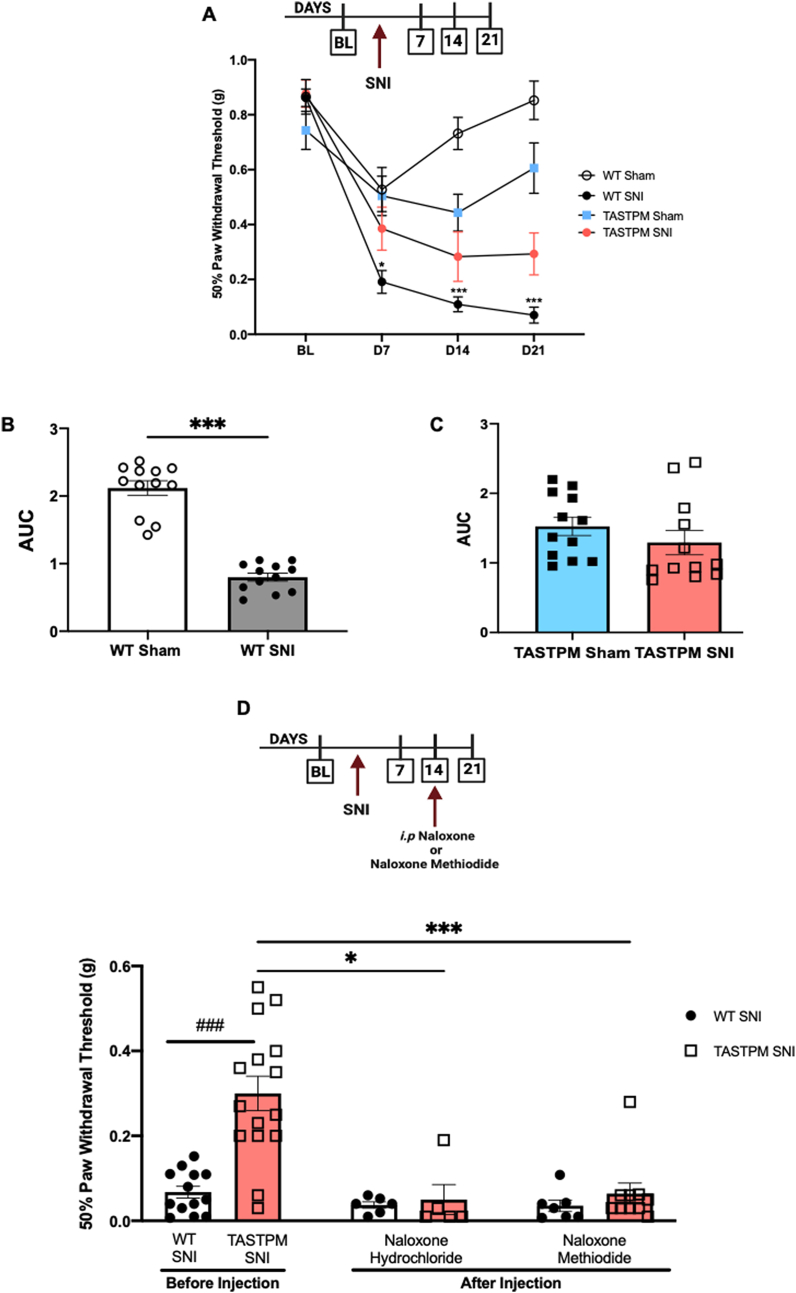
Attenuated mechanical allodynia in TASTPM mice after SNI is associated with an increased opioidergic tone. **(A,B)** Injured WT mice show lower paw withdrawal thresholds (PWT) than sham for 21 days. **(A,C)** Injured TASTPM mice display comparable PWT to sham TASTPM. Data are presented as mean ± SEM (n = 12 mice per group). *p < 0.05 and ***p < 0.001 compared to corresponding sham group, two-way ANOVA repeated measures followed by Tukey test. **(D)** Single *s*ystemic injection of naloxone hydrochloride (1 mg/kg) and naloxone methiodide (1 mg/kg) at day 14 after SNI in WT and TASTPM mice. TASTPM PWT are higher than WT (^###^p < 0.001) before treatments. Values are presented as mean ± SEM (n = 13–15 mice before injection in each group). Mechanical thresholds are reduced in injured TASTPM mice 30 min after naloxone hydrochloride (*p < 0.05) and naloxone methiodide (***p < 0.001). Values are presented as mean ± SEM (n = 5–6 mice per group in naloxone hydrochloride administration and n = 7–10 mice per group in naloxone methiodide administration) *p < 0.05 compared to corresponding group after naloxone hydrochloride administration, ***p < 0.001 compared to corresponding group after naloxone methiodide administration, two-way ANOVA repeated measures followed by Tukey's test.

As expected, in a peripheral nerve injury model of neuropathic pain (SNI (Spared Nerve Injury)), WT paw withdrawal thresholds to mechanical stimulation were significantly lower than sham injury thresholds at 7, 14 and 21 days after injury (Fig. 1 A). Sham surgery resulted in lower thresholds than baseline values at day 7, though thresholds recovered to baseline values at later time points (Fig. 1A). In TASTPM, mechanical thresholds after SNI were close to sham thresholds which were lower than baseline values over a 3 week-period after injury (Fig. 1A). Since in TASTPM we detected a significant sham effect and SNI ipsilateral thresholds showed a trend to be higher than WT, we performed an area under the curve (AUC) analysis. This revealed that WT SNI thresholds were lower than Sham whereas TASTPM SNI thresholds were comparable to Sham (Fig. 1B and C).

Thus, these behavioural data show that TASTPM develop less severe neuropathic allodynia than WT and confirm our previous observations in a model of osteoarthritis pain in which TASTPM recovered from persistent allodynia, a phenomenon that we attributed to involvement of endogenous opioids (Aman et al., 2019). For this reason, here we tested the effect of single systemic administration of either naloxone or naloxone methiodide in neuropathic TASTPM. We selected the 14day SNI time point based on the following evidence: the first was that WT thresholds had recovered from the effect of sham surgery (Fig. 1 A). The second was that before treatments, TASTPM SNI thresholds were significantly less allodynic than WT (Fig. 1D). We observed that the opioid receptor antagonists did not alter WT mechanical thresholds (Fig. 1D). However, in TASTPM both opioid receptor antagonists restored mechanical thresholds to allodynic values comparable to WT thresholds. Specifically, injection of either naloxone or naloxone methiodide brought TASTPM thresholds down to WT values (Fig. 1D).

These results suggest that an inhibitory tone mediated by endogenous opioids contributes to TASTPM partial endurance to the development of neuropathic allodynia. Whilst the CNS is the most obvious area of investigation for opioid mechanisms responsible for such a behaviour in TASTPM, we became intrigued by the possibility that changes in the peripheral immune system may be a contributor factor based of the notions that monocytes/macrophages constitute one of the peripheral sources of opioid peptides (Stein and Lang, 2009; Plein and Rittner, 2018).

### 3.2 Macrophages in TASTPM injured nerve acquire M2-like phenotype

**Fig. 2:**
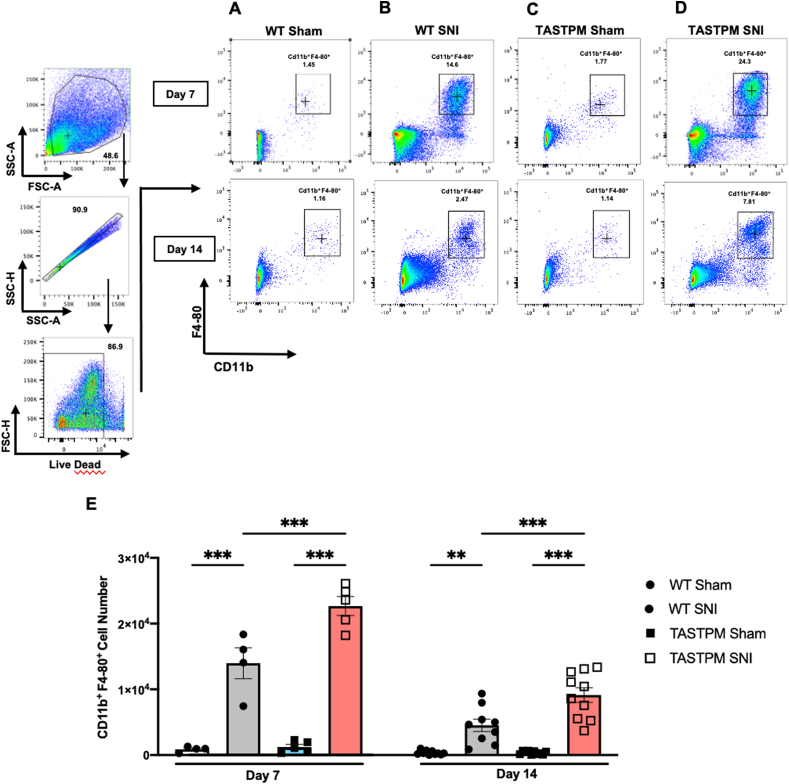
Larger pool of macrophages at TASTPM injury site (A,B,C,D) Gating strategy and representative scatterplots of macrophages in ipsilateral WT and TASTPM sciatic nerves at day 7 and 14 after SNI. Numbers in gates refer to percentage of positive cells for each marker. Cells were gated on F4/80^+^ and CD11b^+^ and macrophages were defined as CD11b^+^F4/80^+^ cells. **(E)** Bar charts represent macrophage (CD11b^+^F4/80^+^) absolute numbers. Data are mean ± S.E.M., N = 4,5 day 7 and N = 9,10/group day 14. *p < 0.05, **p < 0.01 and ***p < 0.001, two-way ANOVA repeated measures followed by Tukey's multiple comparison test.

Considering the mechanistic role played by immune cells at the site of nerve injury in neuropathic pain (Davies et al., 2019; Davoli-Ferreira et al., 2020; Echeverry et al., 2013), we quantified and assessed the phenotype of SNI macrophages in WT and TASTPM using flow cytometry of sciatic nerve cell suspension. As expected, WT macrophage numbers at the site of nerve injury were higher than in sham-injured nerves. Specifically, quantification of the CD11b^+^F4/80^+^ population revealed that numbers peaked at day 7 and slowly declined at day 14 after SNI (Fig. 2 A, B and E). Similarly, in TASTPM injured nerves macrophage numbers were higher than in sham nerves, peaked at day 7 and declined at day 14 (Fig. 2 C, D and E). However, TASTPM macrophages were more abundant than in WT at both day 7 and day 14 after SNI (Fig. 2E) suggesting that more monocytes/macrophages accumulate in TASTPM injured nerves. Of note, we used more mice for the day 14 time points as behavioural experiments with the opioid receptor antagonists were performed at that time point. Nevertheless, we included enough animals (Heneka et al., 2015; Bettcher et al., 2021) in the day 7 experiment for this set of data to bear biological significance.

**Fig. 3:**
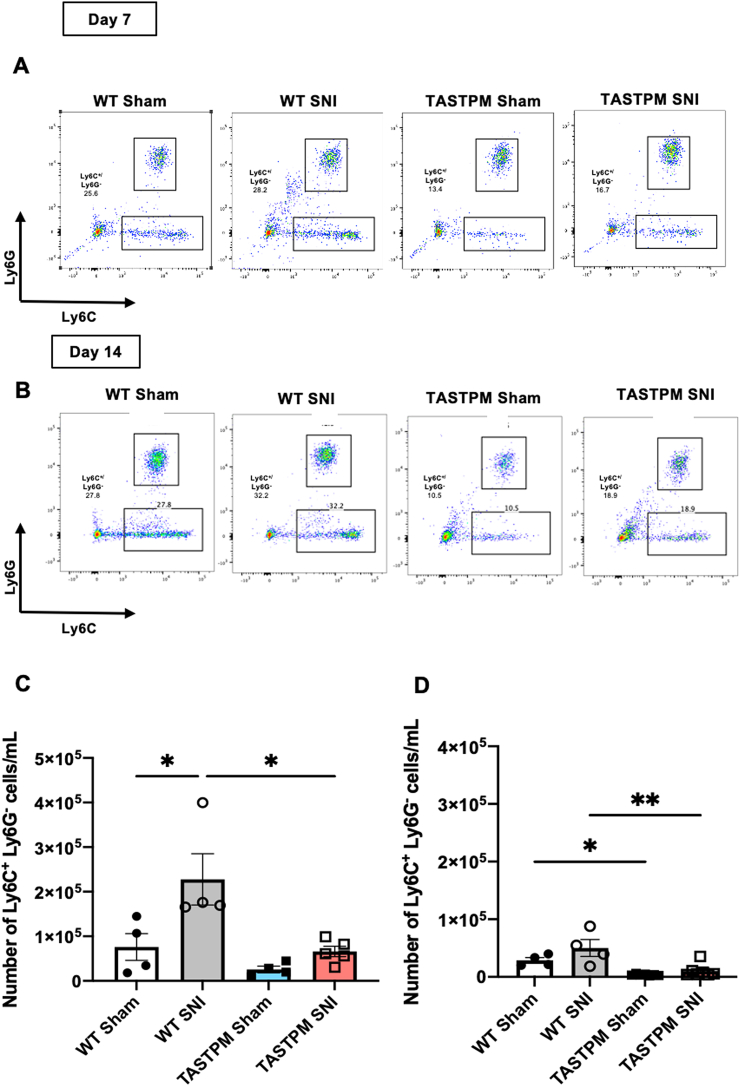
Circulating monocytes (CD11b^+^/Ly6C^+^/Ly6G^−^) are lower in TASTPM. (A,B) Representative scatterplots of circulating monocytes (Ly6C^+^/Ly6G^−^ cells) gated from CD11b^+^ cells from WT and TASTPM at day 7 and 14 after SNI. Numbers in gates refer to percentage of positive cells for each marker. Cells were gated on CD11b and then on Ly6C and Ly6G and circulating monocytes were defined as CD11b^+^/Ly6C^+^/Ly6G^−^. **(C and D)** Bar charts representing number of monocytes (CD11b^+^/Ly6C^+^/Ly6G^−^) at day 7 **(C)** and 14 **(D)** after SNI per ml of blood. Data are mean ± S.E.M., N = 4–7 for each group. *p < 0.05, one-way ANOVA repeated measures followed by Tukey's multiple comparison test.

Although proliferation of local resident macrophages cannot be ruled out, infiltration of monocytes from the periphery occurs in a much larger extent. Thus, we examined blood monocytes at days 7 and 14 after SNI in WT and TASTPM and observed that monocyte numbers and degree of Ly6C expression were genotype- and injury-dependent. Specifically, monocyte numbers (CD11b^+^/LyC6^+^/Ly6G^−^ cells) in WT SNI were higher than in sham at day 7 (Fig. 3A,C and Supplementary Fig. 1A) but not day 14 (Fig. 3B,D) and monocytes were more abundant in WT SNI than TASTPM SNI at day 7 (Fig. 3C). Within the TASTPM groups numbers were comparable at days 7 and 14 after SNI (Fig. 3A–D and Supplementary Fig. 1A). However, TASTPM displayed lower numbers of monocytes than WT under both sham and day 14 SNI conditions (Fig. 3B, D), indicating the occurrence of monocytopenia.

**Fig. 4:**
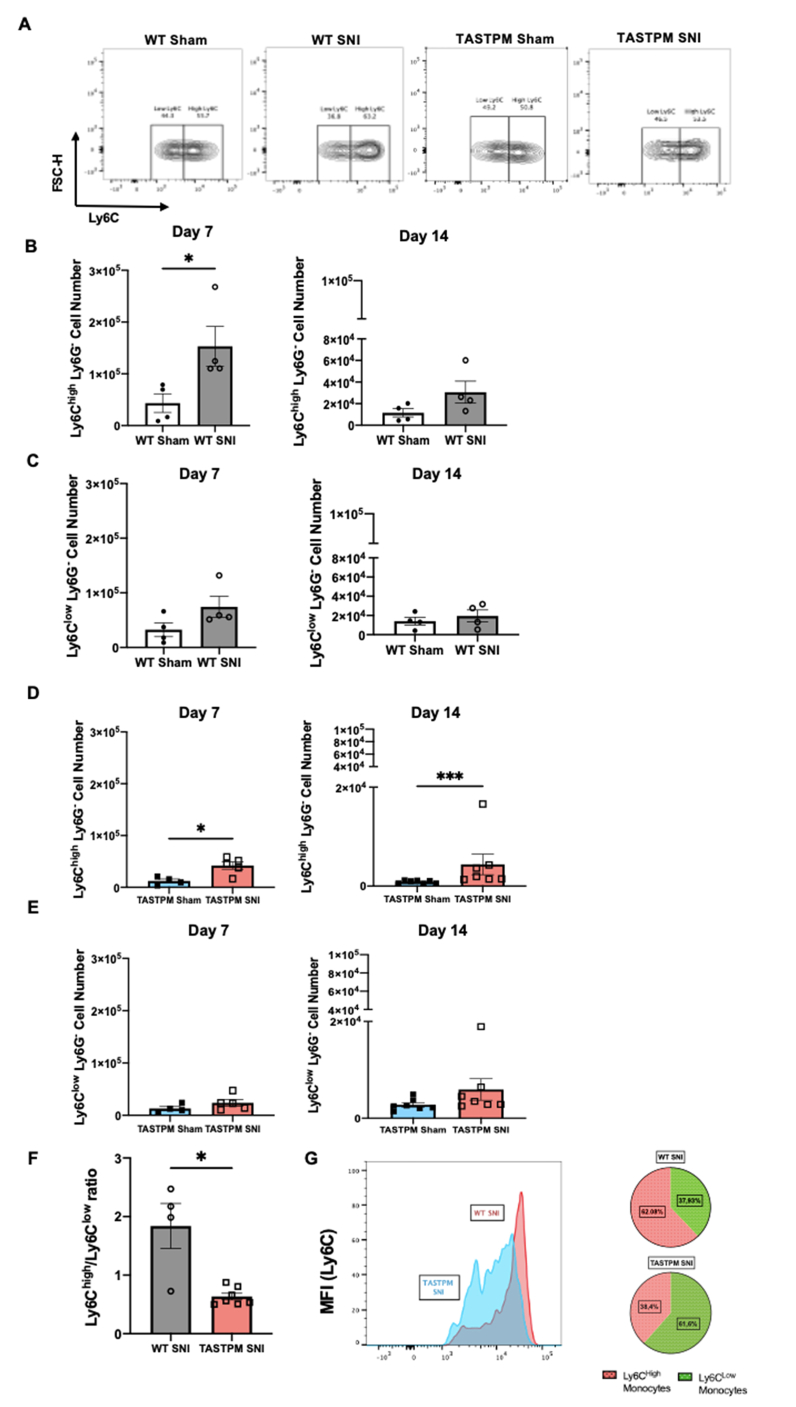
Classical monocytes are less numerous in bloodstream of TASTPM at day 14 after injury. (A) Representative contour plots of circulating Ly6C^low^ and Ly6C^high^ monocytes of WT and TASTPM at day 14 SNI. Numbers in gates refer to the percentage of positive cells for specific Ly6C population. Cells were gated on Ly6C and Ly6G and Ly6C expression was further assessed. **(B, D)** Bar charts representing number of Ly6C^high^ monocytes in WT **(B)** and TASTPM **(D)** mice at day 7 and 14 SNI per ml of blood. **(C,E)** Bar charts representing number of Ly6C^low^ monocytes in WT **(C)** and TASTPM **(E)** mice at days 7 and 14 SNI per ml of blood. **(F)** Bar charts representing circulating Ly6C^high^/Ly6C^low^ ratio at day 14 SNI and representative mean fluorescence intensity (MFI) histogram representing number of Ly6C^high^ and Ly6C^low^ monocytes in WT and TASTPM at day 14 SNI. **(G)** Pie charts indicating lower proportion of Ly6C^high^ monocytes in TASTPM than WT SNI. Data are mean ± S.E.M., N = 4–7 for each group. *p < 0.05, ***p < 0.001, compared to control, Unpaired Student's t-test. Y-axis were adjusted to 3 × 10^5^ and 1 × 10^5^ for better visualization of bar charts representing values.

Taking into consideration that classical monocytes (Ly6C^high^) infiltrate the site of injury in response to inflammatory chemokines (Kalinski et al., 2020), we examined circulating Ly6C^high^ and Ly6C^low^ monocyte numbers in WT and TASTPM at days 7 and 14 after SNI. In WT Ly6C^high^, but not Ly6C^low^ cell numbers were higher in SNI than sham at day 7 (Fig. 4A–C and Supplementary Fig. 1B). Likewise, in TASTPM, Ly6C^high^ but not Ly6C^low^ monocyte numbers were higher in SNI than sham at both days (Fig. 4 A,D,E and Supplementary Fig. 1B).

These data provide evidence for comparable increase of circulating classical monocytes in concomitance to nerve injury in WT and TASTPM and support the notion that classical (Ly6C^high^) monocytes leave the bone marrow and circulate in blood on their way to infiltrate at the injury site (Kratofil et al., 2017). However, whilst in WT at day 14 SNI the distribution of Ly6C^low^ and Ly6C^high^ was in favour of classical monocytes, in TASTPM SNI classical monocytes down numbered non-classical monocytes (Fig. 4F and G). It is tempting to speculate that lower classical monocytes in blood correlate with more macrophages at the injury site in TASTPM.

Altogether these data indicate an intriguing scenario in TASTPM compared to WT whereby TASTPM monocytopenia is associated with less classical monocytes circulating in blood after SNI and higher number of macrophages at the site of nerve injury.

We hypothesised that the nature of the macrophage infiltrate may be different between WT and TASTPM and focused on nerve injury site at day 14 after SNI based on the following considerations: i) TASTPM macrophage numbers were significantly higher than WT, ii) mechanical allodynia was established in WT but less so in TASTPM at this time point and iii) naloxone methiodide revealed the existence of a peripheral inhibitory tone that prevented development of allodynia in TASTPM.

**Fig. 5:**
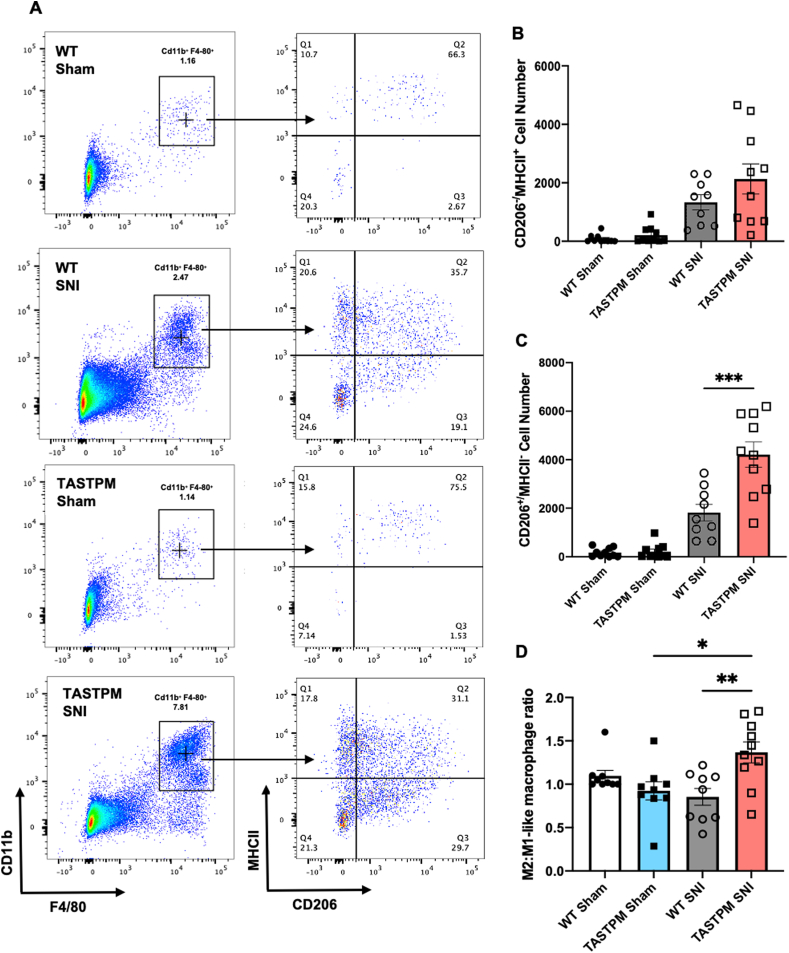
TASTPM macrophages at injury site skew towards a M2-like phenotype (CD206^+^/MHCII^−^). (A) Representative scatterplots of expression of CD206 (M2-like) and MHCII (M1-like) markers in macrophages in ipsilateral sciatic nerves of WT and TASTPM at day 14 SNI. Numbers in gates refer to percentage of positive cells for specific markers. CD11b^+^/F4/80^+^ double positive population identified as macrophages and further evaluated for CD206 and MHCII expression. CD206^+^MHCII^−^ cells were M2-like macrophages and CD206^-^/MHCII^+^ were M1-like macrophages. **(B, C)** Bar charts representing absolute cell numbers of different macrophage population in sciatic nerve. **B)** CD206^-^/MHCII^+^ macrophages and **(C)** CD206^+^/MHCII^−^ macrophages at injury site. **(D)** Bar chart representing the ratio between M2-like macrophages (CD206^+^/MHCII^−^) and M1-like macrophages (CD206^-^/MHCII^+^) at the injury site in WT and TASTPM mice. **(**Data are mean ± S.E.M., N = 9,10 per group. *p < 0.05, **p < 0.01, ***p < 0.001, one-way ANOVA repeated measures followed by Tukey's multiple comparison test.

Specifically, in both WT and TASTPM we found that CD206^-^/MHCII^+^ cells (M1-like macrophages) and CD206^+^/MHCII^−^ cells (M2-like macrophages) accumulated in SNI nerves more than in sham (Fig. 5A–C). However, the M2-like population (CD206^+^/MHCII^−^) was significantly greater in TASTPM than WT at nerve injury site (Fig. 5C and D). We use the M1/M2 like nomenclature for convenience, although we are aware that this is an oversimplification.

These data indicate that at day 14 after SNI M2-like macrophages (reparative) are present in equal number to M1-like macrophages (pro-inflammatory and pro-nociceptive) in WT and support the suggestion that macrophages at the site of injury are not likely to be critical for the maintenance of neuropathic allodynia (Barclay et al., 2007; Yu et al., 2020). Instead, in TASTPM, M2-like macrophages are significantly more abundant than M1-like macrophages and they may contribute to the attenuation of allodynia in TASTPM via anti-nociceptive mechanisms at the nerve injury site (Pannell et al., 2016).

### 3.3 TASTPM macrophages engulf myelin, acquire M2-like phenotype, and overexpress PENK

**Fig. 6:**
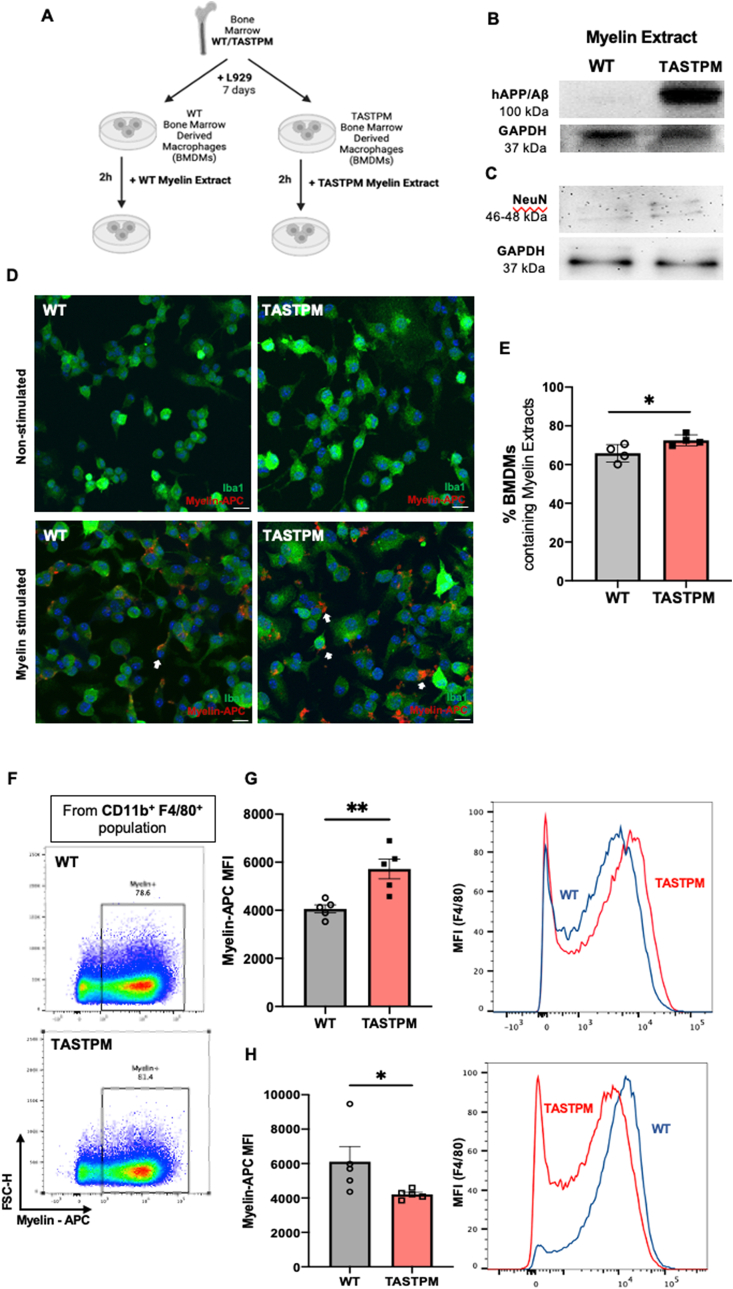
TASTPM bone-marrow derived macrophages (BMDMs) phagocytose more myelin extract containing hAPP/Aβ. (A) Schematic of BMDMs culture and myelin extract stimulation protocol. Created with BioRender.com. **(B)** Myelin extract from TASTPM express the hAPP/Aβ whilst, as expected, myelin extract from WT do not. (**C)** WT and TASTPM Myelin extracts express traces of NeuN. **(D)** Representative images of non-stimulated (PBS only) and myelin stimulated BMDMs from WT and TASTPM. Myelin inclusions in BMDMs (white arrows). Objective x20, scale bar = 20 μm. **(E)** Bar chart representing percentage of WT and TASTPM BMDMs containing myelin extracts after 2 h incubation. Data are mean ± SD, N = 4 coverslips per group. *p < 0.05 unpaired Student's t-test. **(F)** Representative scatterplots of myelin phagocytosis by WT and TASTPM BMDMs. BMDMs defined as F4/80 and CD11b double positive and then gated on labelled myelin. **(G)** Bar chart and representative mean fluorescence intensity (MFI) histogram representing percentage of myelin phagocytosis in WT and TASTPM BMDMs after 2 h incubation. **(H)** Bar chart and representative mean fluorescence intensity (MFI) histogram representing percentage of myelin phagocytosis in WT and TASTPM BMDMs after 2 h incubation with opposite myelin extracts. Data are mean ± S.E.M., N = 3 technical replicates for each group. *p < 0.05, **p < 0.01 unpaired Student's t-test.

With the aim to mimic the site of injury microenvironment and compare WT and TASTPM macrophage phagocytic activity, we prepared bone marrow derived macrophages (BMDMs) from WT and TASTPM and incubated these cells with myelin extracts obtained from either WT or TASTPM brains (Fig. 6A). TASTPM but not WT myelin extracts contained human APP/Abeta (Fig. 6B), and both myelin extracts were promptly taken up by BMDMs after 2 h incubation. These extracts contained traces of neuronal cell bodies using NeuN (Fig. 6C), suggesting that soluble APP/Abeta was present alongside myelin. Quantification of Iba1^+^ cells containing myelin revealed an increased proportion of TASTPM BMDMs containing myelin extracts (Fig. 6D). Quantitative analysis showed increased mean fluorescence intensity (MFI) of myelin expression in TASTPM BMDMs when compared to WT BMDMs (Fig. 6E and F and Supplementary Fig. 2A).

Relevantly, WT BMDMs challenged with TASTPM myelin extracts engulfed myelin in a similar fashion to TASTPM whilst myelin expression in TASTPM BMDMs was reduced after stimulation with WT myelin extracts (Fig. 6H, Supplementary Figs. 2C and D) indicating that the presence of human APP/Abeta in myelin extracts contributes to the phagocytic activity observed in TASTPM macrophages.

**Fig. 7:**
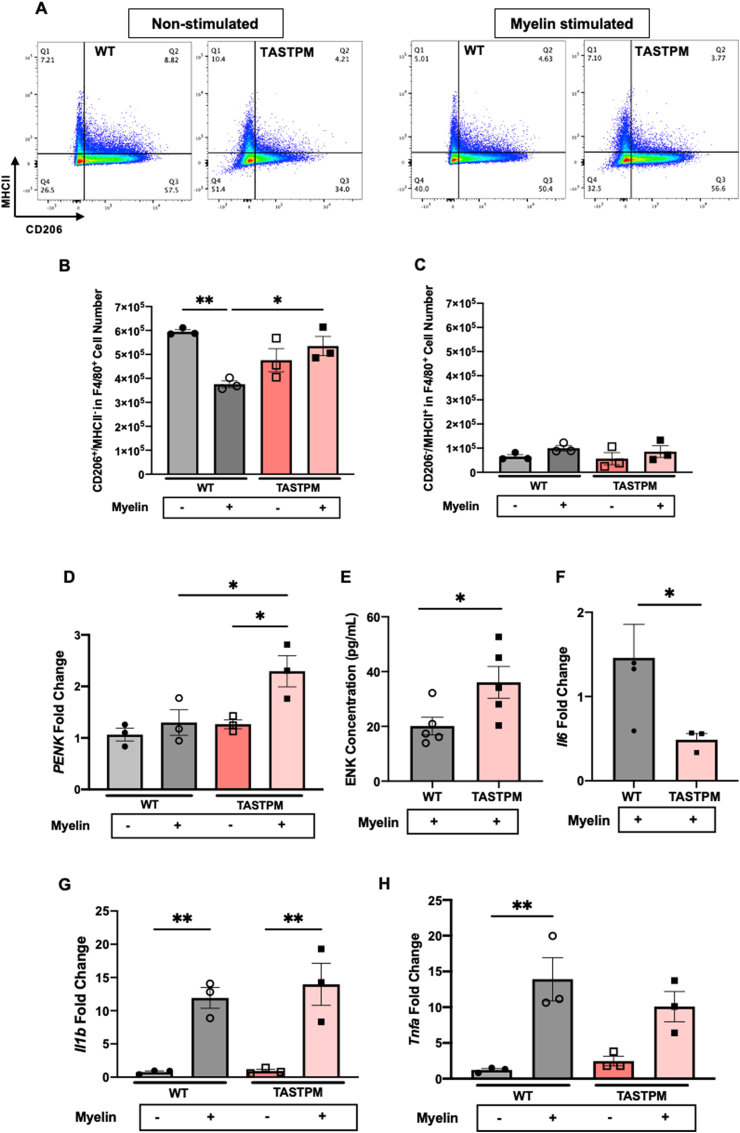
Bone-marrow derived macrophages (BMDMs) from TASTPM acquire M2-like phenotype (CD206^+^/MHCII^−^) and upregulate PENK expression. (A) Representative scatterplots of CD206 (M2-like) and MHCII (M1-like) expression in macrophages from WT and TASTPM following exposure to myelin. Numbers in gates refer to percentage of positive cells for each marker. Cells gated on F4/80 and CD11b and then on CD206 and MHCII. M2-like macrophages were CD206^+^/MHCII^−^ and M1-like macrophages were CD206^-^/MHCII^+^. **(B,C)** Bar charts representing percentage of BMDMs phenotype. **(B)** CD206^+^/MHCII^−^ BMDMs (M2-like) and **(C)** CD206^-^/MHCII^+^ BMDMs (M1-like) after myelin challenge. **(D)** mRNA expression levels for *PENK.* Data are mean ± S.E.M., N = 3 technical replicates for each group. *p < 0.05, **p < 0.01, one-way ANOVA repeated measures followed by Tukey's multiple comparison test. **(E)** Enkephalins content in supernatants from BMDMs after myelin stimulation. Unpaired Student's t-test, *p < 0.05, compared to stimulated WT BMDMs. Data expressed as mean ± SD, N = 5 cultures for each group. **(F)***Il6, Il1β***(G)** and *Tnfα***(H)** mRNA expression levels. Unpaired Student's t-test. Data are mean ± S.E.M., N = 3 technical replicates for each group. *p < 0.05, **p < 0.01 compared to stimulated WT BMDMs.

Following phagocytosis of myelin, the number of WT macrophage (F4/80^+^ cells) which were CD206^+^/MHCII^−^ was lower than in macrophages not exposed to myelin (Fig. 7 A, B). Instead, a higher number of TASTPM macrophages acquired M2-like phenotype after myelin phagocytosis whilst CD206^+^/MHCII^−^ cell numbers were like those not exposed to myelin, and such cells were higher than in WT challenged with myelin (Fig. 7 A, B). CD206^-^/MHCII^+^ (M1-like) macrophage number was higher but not statistically different between WT and TASTPM challenged and not challenged with myelin (Fig. 7C). These data indicate that TASTPM BMDMs avidly engulf myelin extracts that contain human APP/Abeta, a protocol that may resemble the *in vivo* TASTPM situation whereby circulating monocytes accumulate amyloid fragments, and uptake myelin debris once they infiltrate at the injured nerve site.

With a final aim to link these *in vitro* data to behavioural data that indicated a peripheral opioidergic tone in TASTPM, we quantified the expression of mRNA for proenkephalin (*PENK*) in BMDMs cell lysates and enkephalins content in culture media. We found that *PENK* was significantly upregulated in TASTPM BMDMs stimulated with TASTPM myelin extracts compared to WT and TASTPM BMDMs unstimulated (Fig. 7D). Furthermore, enkephalins levels were higher in TASTPM than WT BMDMs supernatants stimulated with myelin extracts (Fig. 7E). Consistently, mRNA for the pronociceptive cytokine IL-6 was downregulated in TASTPM BMDMs stimulated with TASTPM myelin compared to WT (Fig. 7F). However, *Il1β* and *tnfα* were upregulated in both WT and TASTPM BMDMs after myelin challenge (Fig. 7G and H), likely representing the proportion of BMDMs which acquire M1-like phenotype, whilst *Ccr2* and *Tgfb* expression was not significantly altered (Supplementary Figs. 3A–B).

Altogether these *in vitro* data suggest that in TASTPM *in vivo* scenario macrophages which derive from monocytes that contain amyloid fragments would promptly phagocyte myelin at the site of injury, acquire M2-like phenotype and release enkephalins which inhibit nociceptive neuron activity via activation of opioid receptors.

## 4 Discussion

This study shows that TASTPM transgenic AD mice are less susceptible to the development of neuropathic allodynia due to high opioid inhibitory tone, that, in the periphery, is mediated by infiltrating immune cells, mainly macrophages, at the site of nerve injury. Indeed, these immune cells can release opioid peptides which inhibit nociceptive signalling via activation of opioid receptors located in nociceptors (Stein and Lang, 2009; Plein and Rittner, 2018).

We have previously reported that 6-month-old TASTPM mice present AD-related pathology in key regions of the pain pathway including amyloid plaques in the cortex, hippocampus, and thalamus which are surrounded by barriers formed by astrocytes and microglia. In the spinal cord, amyloid deposits are detected in 12-month-old TASTPM whereas 6-month-old TASTPM dorsal horn neurons display intracellular APP/Abeta expression, high levels of pre–pro (*pPENK*) and proenkephalin (*PENK*) mRNA and enkephalin peptide. At six months of age, TASTPM mice exhibit cognitive deficits and an age-dependent decline in sensitivity to thermal stimulation that is reversed by the opioid receptor antagonist naloxone (Aman et al., 2016). Indeed, in TASTPM mice there is a significant inhibitory opioidergic tone, which becomes evident under persistent pain conditions and promotes recovery from allodynia in a model of osteoarthritis pain (Aman et al., 2019). We have now strengthened the relevance of such an inhibitory tone alteration and report that in a model of neuropathic pain TASTPM mice develop only partial allodynia and show complete allodynia after treatment with either centrally penetrant- or peripheralized-naloxone. We suggest an increase of the opioidergic tone, including a significant peripheral component, as a contributing factor to TASTPM mice resistance to show neuropathic pain-like behaviour. We do not rule out central mechanisms of endogenous opioid-mediated analgesia in TASTPM but focused on possible peripheral tone. Likewise, we speculate that TASTPM might show attenuated responses in other pain behaviour modalities such as spontaneous pain.

Endogenous opioids are expressed by CNS neurons at both spinal and supraspinal levels and by immune cells such as macrophages in the periphery, where they act on neuronally expressed opioid receptors. Opioids are first line of treatment for severe pain but liable to abuse and overdose. Such problematic CNS side effects have prompted to consider affecting opioid receptors expressed by peripheral sensory neurons as analgesic targets. For instance, endogenous opioids released from immune cells at the site of nerve injury attenuate allodynia in neuropathic pain models (Celik et al., 2016; Labuz et al., 2009). Furthermore, adoptive transfer of M2 macrophages injected perineurally attenuates neuropathic allodynia (Pannell et al., 2016).

In this study, in neuropathic TASTPM mice, we have identified anti-nociceptive monocytes/macrophages that accumulate at the site of nerve injury and display M2-like (CD206^+^/MHCII^−^) phenotype. Whether macrophage phenotype is altered at the cell bodies of sensory neurons in the DRG remains to be explored. Following peripheral nerve injury, circulating monocytes are recruited to the injured nerve a few days after injury, continue to infiltrate for several weeks and engraft in the pool of resident macrophages (Ydens et al., 2020; Taskinen and Roytta, 1997). Several reports have indicated that blood borne Ly6C^high^ (classical) monocytes are robustly recruited into the injury site and undergo *in situ* differentiation (Geissmann et al., 2010). However, Ly6C^low^ (non-classical) monocytes, well-known for patrolling the endothelium, also extravasate from the bloodstream into injured tissue (Old et al., 2014).

As reported by others, monocytes are critical for the initiation of neuropathic pain (Peng et al., 2016). However, pro-inflammatory monocytes/macrophages that accumulate at the site of injury may not play a significant pro-nociceptive role (Yu et al., 2020). Instead, the polarization of macrophages towards a M2-like phenotype by application of IL-4 to the injured nerve results in significant anti-allodynic effect through production and release of endogenous opioids (Pannell et al., 2016). Similarly, in TASTPM injured nerve, accumulation of CD206^+^/MHCII^−^ macrophages correlates with attenuation of neuropathic allodynia and with an increased frequency of Ly6C^low^ monocytes in the TASTPM bloodstream. Furthermore, TASTPM BMDMs skew toward a CD206^+^/MHCII^−^ phenotype upon myelin phagocytosis, upregulate *PENK* mRNA and accumulate extracellular enkephalins (schematic in Supplementary Fig. 4).

We argue that for such a behaviour to be adopted in TASTPM *in vivo*, the most plausible explanation is that presence of amyloid peptides in blood and their interaction with monocytes/macrophages alters Ly6C expression and monocyte recruitment, ultimately leading to an increased phagocytosis of myelin debris by these immune cells at the site of nerve injury. Several reports have shown increased phagocytic activity of bone-marrow derived macrophages from mice with AD pathology upon stimulation with amyloid-β (Li et al., 2020; Koronyo et al., 2015). Our *in vitro* model mimics the *in vivo* situation where circulating monocytes engulf amyloid-β peptides that outflow from the brain in TASTPM mice (Halle et al., 2015) and predicts a mechanism whereby infiltrating macrophages derived from such monocytes behave in a unique manner at the site of nerve injury. Here they are likely to perform efficient phagocytosis, up-regulate enkephalin mRNA expression and release enkephalins that inhibit nociceptive neuron activity. Our proposal for such a unique biology of circulating monocytes is in line with existing evidence in APP_swe_/PS1 transgenic AD mice which, like TASTPM, display monocytopenia and low number of circulating classical monocytes (Naert and Rivest, 2012, 2013). Additionally, monocytopenia is also present in AD patients (Lunnon et al., 2012) and CCR2, a critical receptor expressed by Ly6C^high^ monocytes, is shown to be decreased in monocytes of AD patients (Zhang et al., 2013).

The concept that peripheral and central immune systems are in regular communication in AD has emerged from clinical studies (Bettcher et al., 2021). Despite strong clinical implications, how peripheral immune responses are altered in AD and how these can influence the development of neuropathic pain remains enigmatic. This notion has led our investigation into peripheral immune mechanisms, which are a feature of neuropathic pain, in TASTPM mice, which recapitulate some key features of AD brain pathology as well as circulating monocytes containing myeloid peptides (Halle et al., 2015).

Our main goal was to understand how the peripheral immune system, altered by AD pathology, contributes to a distinct peripheral immune response in chronic neuropathic pain conditions. In TASTPM, human APP is expressed primarily in neurons. However, in this animal model and others, pathological amyloid-beta can be detected in plasma (Halle et al., 2015) and there is evidence for the efflux of amyloid-beta generated in the brain to blood circulation (Storck et al., 2016). We argue that the presence of these amyloid peptides in blood can promote changes in immune cells, which upon nerve injury, can differentially respond and contribute to an altered monocyte phenotype and, consequently, blood-borne macrophage phenotype. In our *in vitro* experiments, we generated myelin extracts, which contained traces of neuronal cells, which may account for the detection of APP/Abeta in the myelin extracts. By stimulating our BMDMs with it, we aimed to mimic the *in vivo* scenario, as sciatic nerve injury leads to the generation of myelin fragments, cellular debris and disruption of blood-nerve barrier. This results in the release of ENK release by TASTPM BMDMs, which suggests that such conditions promote BMDMs to release opioids.

Our behavioural data are pool of male and female data points since we observed no sexual dimorphism. However, we cannot rule out the effect of sex on immune cells under neuropathic pain and neurodegenerative conditions.

Current pain management and treatment in individuals with dementia remains unsatisfactory. Opioids, especially transdermal buprenorphine patches or fentanyl, are frequently prescribed to AD patients than elderly without AD (Bullock et al., 2019; Griffioen et al., 2019).

In conclusion, our data have provided compelling evidence for an increased peripheral opioidergic tone and attenuated neuropathic allodynia, mediated by peripheral immune cells, in our model of AD. These results highlight the need for a tailored therapeutic approach for pain in AD people and support strategies designed to promote polarization of macrophages towards M2-like phenotype for the relief of neuropathic pain (Pannell et al., 2016; Kiguchi et al., 2015).

## Availability of data and materials

The datasets used and/or analysed during the current study are available from the corresponding author on a reasonable request.

## Funding

This work was supported by the European Union's Horizon 2020 Research and Innovation Programme under the Marie Skłodowska-Curie Grant Agreement No 764860 (TOBeATPAIN; RS, GS-L, MM) and from MRC MICA award MR/T002883/1 (LZ, SF, MM).

## Author contributions

Conception/design: RS, LZ, MM; experimental work/data acquisition/analysis: RS, LZ, GS, SF; interpretation of data: RS, LZ, MM; Manuscript writing - original draft: MM; Manuscript revision/editing: RS, MM; All authors read and approved the final manuscript.

## Declaration of competing interest

The authors declare that they have no known competing financial interests or personal relationships that could have appeared to influence the work reported in this paper.

## Data Availability

Data will be made available on request.
