# Supplementary material for: CD206+/MHCII− macrophage accumulation at nerve injury site correlates with attenuation of allodynia in TASTPM mouse model of Alzheimer's disease
Source: Brain Behav Immun Health. 2022 Nov 1;26:100548. doi: 10.1016/j.bbih.2022.100548 (PMC9643400; doi:10.1016/j.bbih.2022.100548)
Supplement: Multimedia component 1 [file mmc1.pdf]

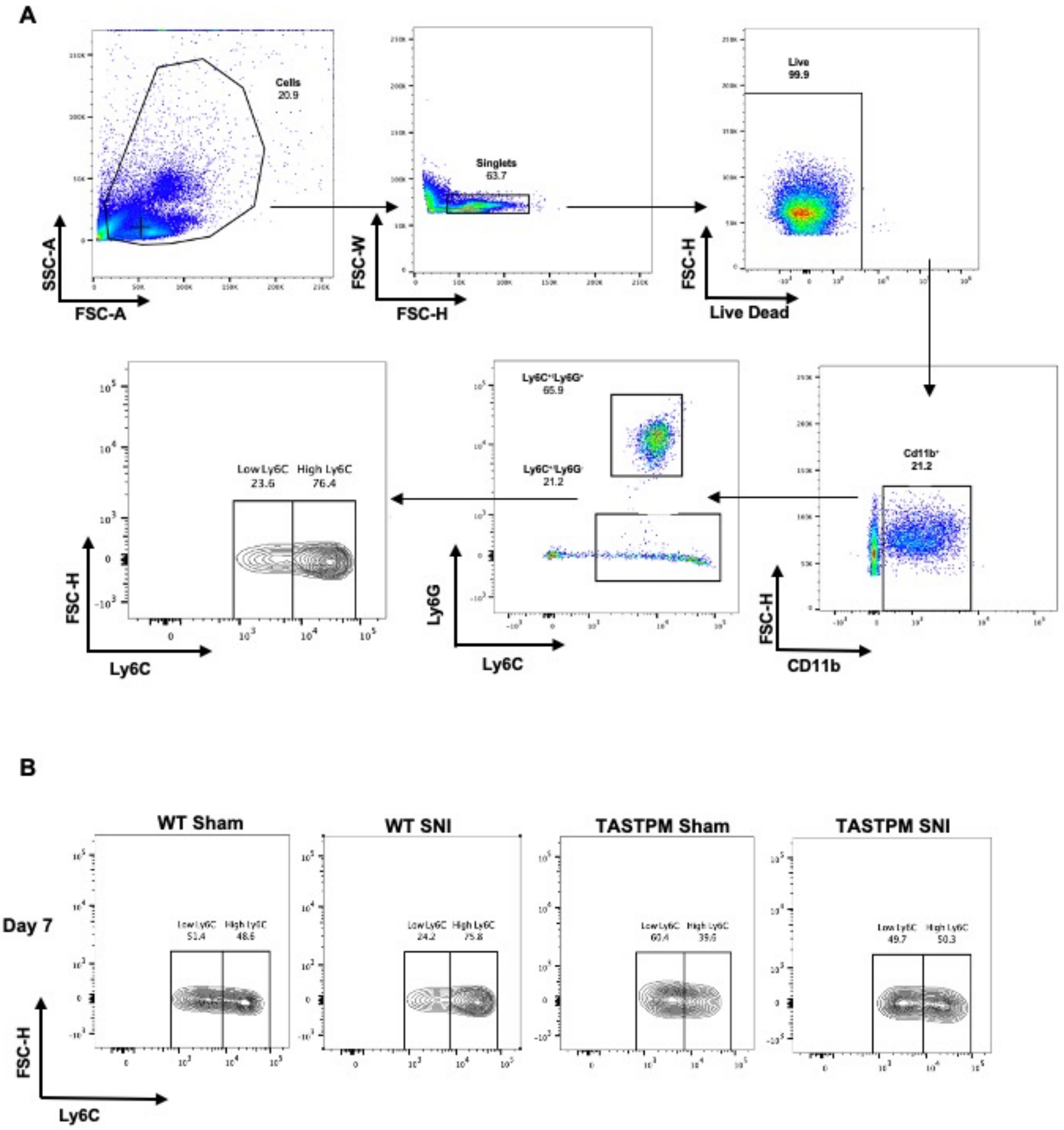

Supplementary Figure 1: **(A)** Gating strategy utilized for the detection of circulating monocytes ($CD11b^+/Ly6C^+/Ly6G^-$). **(B)** Representative contour plots of circulating Ly6C^{low} and Ly6C^{high} from peripheral blood obtained from WT mice or TASTPM mice on day 7 after SNI injury. Numbers in gates refer to the percentage of positive cells for each specific Ly6C population. Cells were gated on Ly6C and Ly6G and Ly6C expression was further assessed.

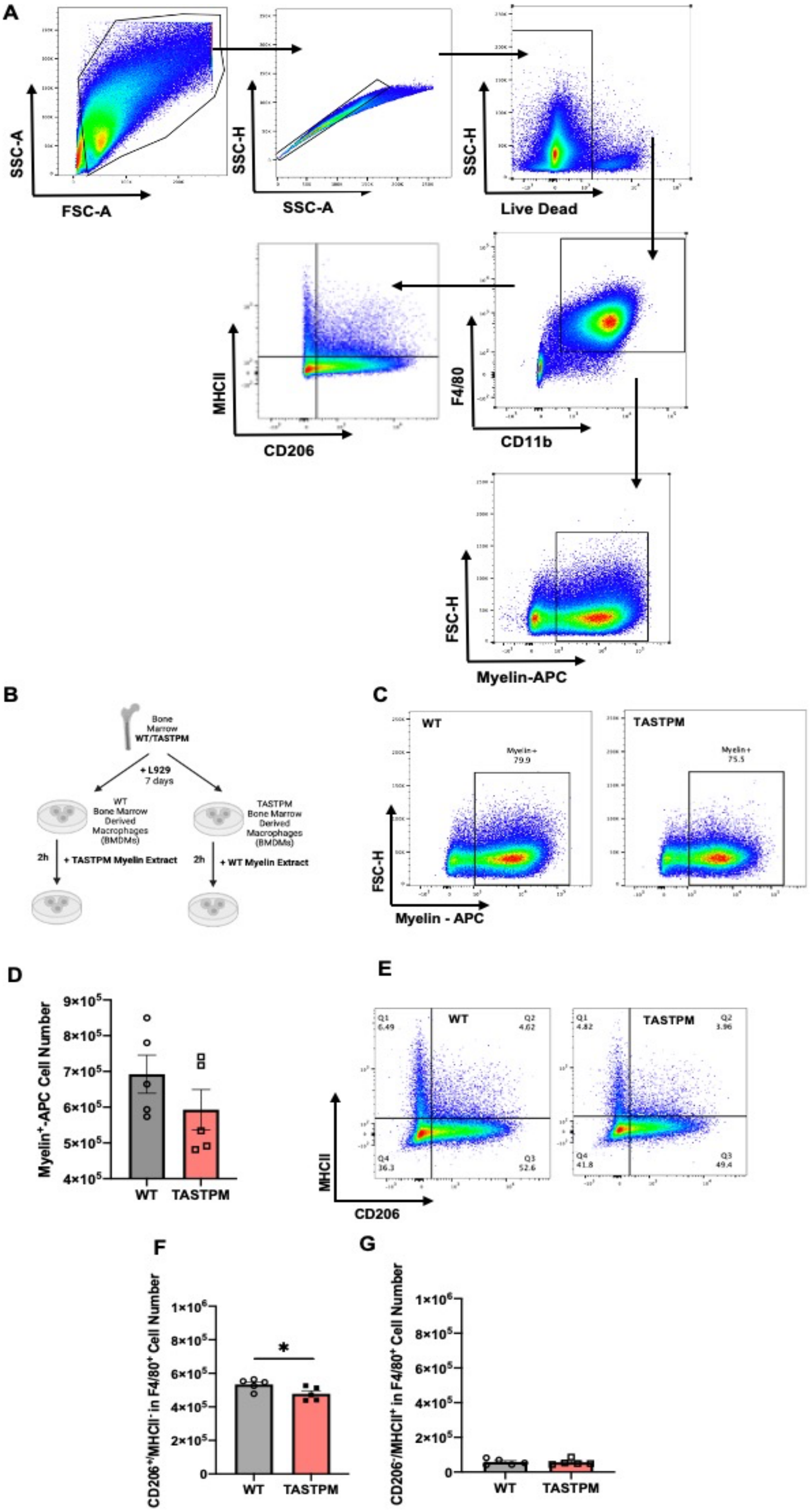
A
SSC-A
FSC-A
SSC-H
SSC-A
SSC-H
Live Dead
MHCII
CD206
F4/80
CD11b
FSC-H
Myelin-APC
B
Bone Marrow WT/TASTPM
+ L929 7 days
WT Bone Marrow Derived Macrophages (BMDMs)
TASTPM Bone Marrow Derived Macrophages (BMDMs)
2h
+ TASTPM Myelin Extract
2h
+ WT Myelin Extract
C
WT
Myelin+ 79.9
TASTPM
Myelin+ 75.5
FSC-H
Myelin - APC
D
Myelin+-APC Cell Number
9×10^5
8×10^5
7×10^5
6×10^5
5×10^5
4×10^5
WT
TASTPM
E
WT
Q1 6.49
Q2 4.62
Q4 36.3
Q3 52.6
TASTPM
Q1 4.82
Q2 3.96
Q4 41.8
Q3 49.4
MHCII
CD206
F
CD206+/MHCII- in F4/80+ Cell Number
1×10^6
8×10^5
6×10^5
4×10^5
2×10^5
0
*
WT
TASTPM
G
CD206-/MHCII+ in F4/80+ Cell Number
1×10^6
8×10^5
6×10^5
4×10^5
2×10^5
0
WT
TASTPM

Supplementary Figure 2: **(A)** Gating strategy used for the detection of myelin and bone-marrow derived macrophage phenotype following myelin challenge. **(B)** Schematic of BMDMs culture and myelin extract stimulation protocol from different donors. Created with BioRender.com. **(C)** Representative scatterplots of myelin phagocytosis mediated by WT and TASTPM BMDMs. BMDMs were defined as F4/80$^+$ and CD11b$^+$ double positive and then gated on labelled myelin from different donors. **(D)** Bar chart representing the number of BMDMs containing fluorescent-labeled myelin in WT and TASTPM BMDMs after 2 hours of incubation. **(E)** Representative scatterplots of expression of CD206 (M2-like) and MHCII (M1-like) receptors in BMDMs from WT mice or TASTPM mice following myelin exposure. Numbers in gates refer to the percentage of positive cells for each specific marker. Cells were gated on F4/80 and CD11b and then on CD206 and MHCII. M2-like macrophages were considered to be CD206$^+$/MHCII$^-$ whilst M1-like macrophages were referred to as CD206$^-$/MHCII$^+$. **(F,G)** Bar charts representing the number of BMDMs with distinct phenotypes. **(F)** CD206$^+$/MHCII$^-$ BMDMs (M2-like) and **(G)** CD206$^-$/MHCII$^+$ BMDMs (M1-like) after opposite myelin challenge. Data expressed as mean ± SD, N=5 cultures for each group, Unpaired Student's t-test.

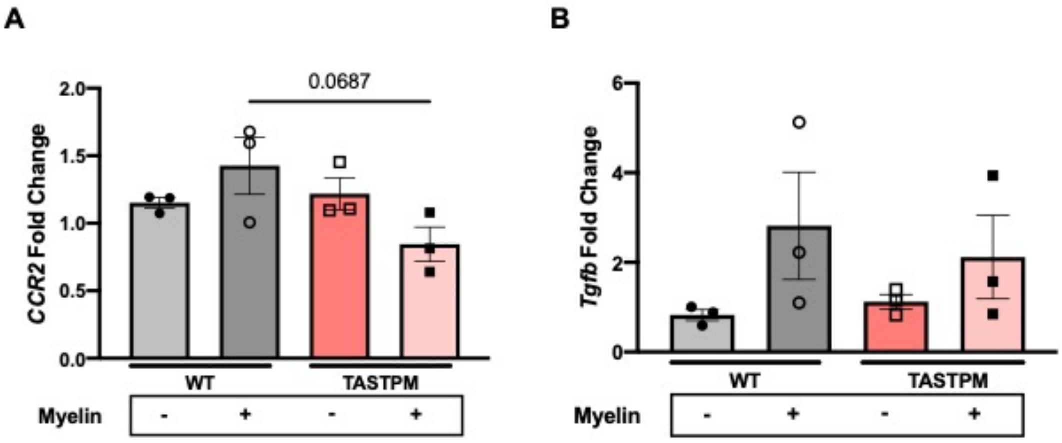

Supplementary Figure 3: mRNA expression levels for *Ccr2* **(A)** and *Tgfb* **(B)** following BMDMs challenge with myelin. Data are mean ± S.E.M., N=3 technical replicates for each group. One-way ANOVA repeated measures followed by Tukey's multiple comparison test.

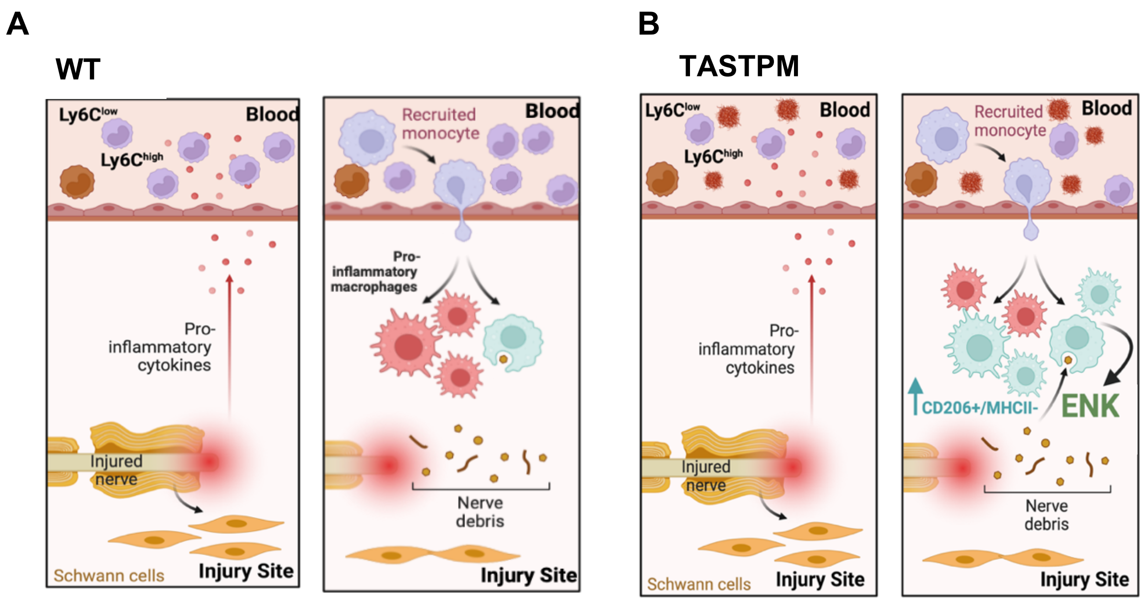

Supplementary Figure 4: An altered peripheral immune response contributes to attenuated neuropathic allodynia in TASTPM mice. **(A)** In WT following nerve injury, proinflammatory cytokines/chemokines are released by cells at the injury site, where they promote extravasation of circulating immune cells, such as (Ly6C^{high}) monocytes. At 14 days following injury, accumulated macrophages have pro- and anti-inflammatory profiles in WT. **(B)** In TASTPM the presence of amyloid peptides in blood may influence monocyte recruitment and macrophage phenotype at the injury site. Indeed, M2-like macrophages are found in higher number at TASTPM injury site where they likely release enkephalins, attenuating neuropathic allodynia 14 days following injury. Created with BioRender.com
